# 1,3-Bis(4-chloro­phen­yl)-4,5-diethoxy­imidazolidine

**DOI:** 10.1107/S1600536808031176

**Published:** 2008-10-22

**Authors:** Yu Wan, Xiu-mei Chen, Pu Zhang, Hui Wu

**Affiliations:** a School of Chemistry and Chemical Engineering, Xuzhou Normal University, Xuzhou, Jiangsu 221116, People’s Republic of China

## Abstract

In the mol­ecule of the title compound, C_19_H_22_Cl_2_N_2_O_2_, the two benzene rings are oriented at a dihedral angle of 3.70 (3)°. The five-membered ring adopts an envelope conformation.

## Related literature

For general background, see: Bunnage & Owen (2008 ▶); Weinreb (2007 ▶); Jin (2006 ▶); Farnia *et al.* (1997 ▶); Reed & Schleyer (1988 ▶). For bond-length data, see: Allen *et al.* (1987 ▶).
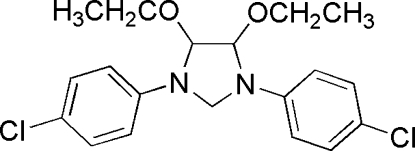


## Experimental

### 

#### Crystal data

C_19_H_22_Cl_2_N_2_O_2_
*M* *_r_* = 381.29
Monoclinic, 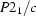

*a* = 10.928 (2) Å
*b* = 11.123 (2) Å
*c* = 16.006 (3) Å
β = 94.480 (15)°
*V* = 1939.6 (6) Å^3^
*Z* = 4
Mo *K*α radiation
μ = 0.35 mm^−1^
*T* = 296 (2) K
0.50 × 0.42 × 0.22 mm

#### Data collection

Siemens *P*4 diffractometer
Absorption correction: multi-scan (*SADABS*; Sheldrick, 1996 ▶) *T*_min_ = 0.843, *T*_max_ = 0.926
4160 measured reflections
3609 independent reflections
1867 reflections with *I* > 2σ(*I*)
*R*_int_ = 0.010
3 standard reflections every 97 reflections intensity decay: 1.1%

#### Refinement

*R*[*F* ^2^ > 2σ(*F* ^2^)] = 0.039
*wR*(*F* ^2^) = 0.072
*S* = 0.94
3609 reflections
227 parameters
H-atom parameters constrained
Δρ_max_ = 0.12 e Å^−3^
Δρ_min_ = −0.13 e Å^−3^

### 

Data collection: *XSCANS* (Siemens, 1996 ▶); cell refinement: *XSCANS*; data reduction: *XSCANS*; program(s) used to solve structure: *SHELXS97* (Sheldrick, 2008 ▶); program(s) used to refine structure: *SHELXL97* (Sheldrick, 2008 ▶); molecular graphics: *SHELXTL* (Sheldrick, 2008 ▶); software used to prepare material for publication: *SHELXTL*.

## Supplementary Material

Crystal structure: contains datablocks global, I. DOI: 10.1107/S1600536808031176/hk2538sup1.cif

Structure factors: contains datablocks I. DOI: 10.1107/S1600536808031176/hk2538Isup2.hkl

Additional supplementary materials:  crystallographic information; 3D view; checkCIF report

## Figures and Tables

**Fig. 1.**
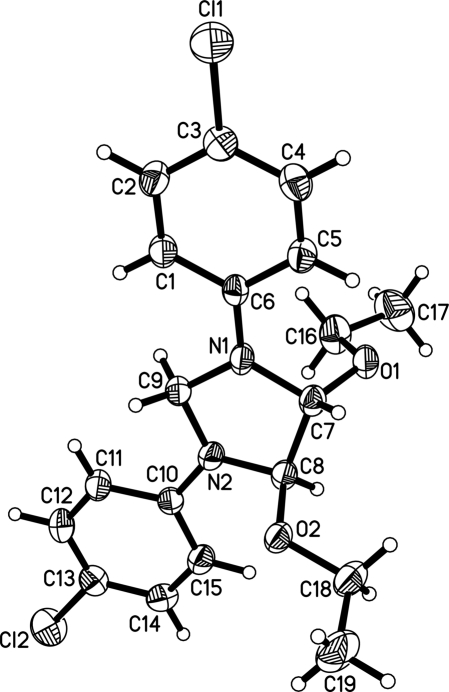
The molecular structure of the title molecule, with the atom-numbering scheme. Displacement ellipsoids are drawn at the 30% probability level.

## supplementary crystallographic information

### Comment

Imidazoles are commonly utilized substructures within the pharmaceutical industry, as these heterocycles impart unique physical and biological properties to compounds of interest (Bunnage & Owen, 2008; Weinreb, 2007; Jin, 2006). Furthermore, molecules containing anomeric effect in N—C—N system influences many structural and electronic properties (Farnia *et al.*,  1997; Reed & Schleyer, 1988). We report herein the synthesis and crystal structure of the title compound.

In the molecule of the title compound (Fig. 1), the bond lengths (Allen *et al.*,  1987) and angles are within normal ranges. Rings A (C1-C6) and C (C10-C15) are, of course, planar and the dihedral angle between them is 3.70 (3)°. So, they are also nearly coplanar. Ring B (N1/N2/C7-C9) is not planar, and adopts envelope conformation with C8 atom displaced by 0.336 (3) Å from the plane of the other ring atoms.

### Experimental

The title compound was simply prepared by the reaction of 4-chloro- benzaldehyde (2.0 mmol) with glyoxal(1.0 mmol) and formaldehyde (1.0 mmol) in ethanol (3.0 ml) at 273–278 K without catalyst for 40 h (yield; 81%). Single crystals suitable for X-ray analysis were obtained from an ethanol solution by slow evaporation.

### Refinement

H atoms were positioned geometrically, with C-H = 0.93, 0.98, 0.97 and 0.96 Å for aromatic, methine, methylene and methyl H, respectively, and constrained to ride on their parent atoms with U_iso_(H) = xU_eq_(C), where x = 1.5 for methyl H and x = 1.2 for all other H atoms.

### Crystal data

**Table:**

C_19_H_22_Cl_2_N_2_O_2_	*F*(000) = 800
*M**_r_* = 381.29	*D*_x_ = 1.306 Mg m^−^^3^
Monoclinic, *P*2_1_/*c*	Melting point = 445–446 K
Hall symbol: -P 2ybc	Mo *K*α radiation, λ = 0.71073 Å
*a* = 10.928 (2) Å	Cell parameters from 28 reflections
*b* = 11.123 (2) Å	θ = 4.9–14.4°
*c* = 16.006 (3) Å	µ = 0.35 mm^−^^1^
β = 94.480 (15)°	*T* = 296 K
*V* = 1939.6 (6) Å^3^	Block, colorless
*Z* = 4	0.50 × 0.42 × 0.22 mm

### Data collection

**Table:**

Siemens P4 diffractometer	1867 reflections with *I* > 2σ(*I*)
Radiation source: normal-focus sealed tube	*R*_int_ = 0.010
graphite	θ_max_ = 25.5°, θ_min_ = 1.9°
ω scans	*h* = 0→13
Absorption correction: multi-scan (SADABS; Sheldrick, 1996)	*k* = 0→13
*T*_min_ = 0.843, *T*_max_ = 0.926	*l* = −19→19
4160 measured reflections	3 standard reflections every 97 reflections
3609 independent reflections	intensity decay: 1.1%

### Refinement

**Table:**

Refinement on *F*^2^	Secondary atom site location: difference Fourier map
Least-squares matrix: full	Hydrogen site location: inferred from neighbouring sites
*R*[*F*^2^ > 2σ(*F*^2^)] = 0.039	H-atom parameters constrained
*wR*(*F*^2^) = 0.072	*w* = 1/[σ^2^(*F*_o_^2^) + (0.0226*P*)^2^] where *P* = (*F*_o_^2^ + 2*F*_c_^2^)/3
*S* = 0.94	(Δ/σ)_max_ = 0.001
3609 reflections	Δρ_max_ = 0.12 e Å^−^^3^
227 parameters	Δρ_min_ = −0.13 e Å^−^^3^
0 restraints	Extinction correction: SHELXL97 (Sheldrick, 2008), Fc^*^=kFc[1+0.001xFc^2^λ^3^/sin(2θ)]^-1/4^
Primary atom site location: structure-invariant direct methods	Extinction coefficient: 0.0056 (4)

### Special details

**Table:**

Geometry. All e.s.d.'s (except the e.s.d. in the dihedral angle between two l.s. planes) are estimated using the full covariance matrix. The cell e.s.d.'s are taken into account individually in the estimation of e.s.d.'s in distances, angles and torsion angles; correlations between e.s.d.'s in cell parameters are only used when they are defined by crystal symmetry. An approximate (isotropic) treatment of cell e.s.d.'s is used for estimating e.s.d.'s involving l.s. planes.
Refinement. Refinement of *F*^2^ against ALL reflections. The weighted *R*-factor *wR* and goodness of fit *S* are based on *F*^2^, conventional *R*-factors *R* are based on *F*, with *F* set to zero for negative *F*^2^. The threshold expression of *F*^2^ > σ(*F*^2^) is used only for calculating *R*-factors(gt) *etc*. and is not relevant to the choice of reflections for refinement. *R*-factors based on *F*^2^ are statistically about twice as large as those based on *F*, and *R*- factors based on ALL data will be even larger.

### Fractional atomic coordinates and isotropic or equivalent isotropic displacement parameters (Å^2^)

**Table:**

	*x*	*y*	*z*	*U*_iso_*/*U*_eq_	
Cl1	0.87517 (6)	−0.07126 (6)	0.61089 (5)	0.0967 (3)	
Cl2	0.24717 (6)	0.95415 (6)	0.65343 (4)	0.0981 (3)	
O1	0.92261 (12)	0.55731 (13)	0.57515 (9)	0.0596 (4)	
O2	0.66798 (11)	0.63533 (12)	0.42946 (8)	0.0586 (4)	
N1	0.73769 (14)	0.43816 (15)	0.56395 (10)	0.0544 (5)	
N2	0.63188 (15)	0.61147 (15)	0.57001 (10)	0.0547 (5)	
C1	0.69410 (19)	0.23950 (19)	0.61290 (13)	0.0576 (6)	
H1	0.6210	0.2672	0.6320	0.069*	
C2	0.7256 (2)	0.1204 (2)	0.62321 (13)	0.0628 (6)	
H2	0.6740	0.0681	0.6493	0.075*	
C3	0.8326 (2)	0.07892 (19)	0.59514 (14)	0.0601 (6)	
C4	0.9094 (2)	0.1553 (2)	0.55715 (14)	0.0669 (7)	
H4	0.9820	0.1264	0.5380	0.080*	
C5	0.8794 (2)	0.2745 (2)	0.54722 (13)	0.0605 (6)	
H5	0.9325	0.3263	0.5222	0.073*	
C6	0.76964 (18)	0.31880 (19)	0.57444 (12)	0.0481 (5)	
C7	0.81176 (18)	0.52749 (18)	0.52714 (12)	0.0520 (6)	
H7	0.8327	0.4979	0.4724	0.062*	
C8	0.72362 (17)	0.63322 (18)	0.51288 (12)	0.0510 (5)	
H8	0.7660	0.7093	0.5262	0.061*	
C9	0.62711 (17)	0.48661 (18)	0.59411 (12)	0.0517 (6)	
H9A	0.6272	0.4782	0.6544	0.062*	
H9B	0.5548	0.4474	0.5677	0.062*	
C10	0.54049 (18)	0.6921 (2)	0.58676 (12)	0.0482 (5)	
C11	0.44177 (18)	0.6556 (2)	0.63022 (13)	0.0587 (6)	
H11	0.4354	0.5755	0.6458	0.070*	
C12	0.3535 (2)	0.7359 (2)	0.65051 (14)	0.0666 (7)	
H12	0.2884	0.7096	0.6798	0.080*	
C13	0.3603 (2)	0.8540 (2)	0.62809 (13)	0.0613 (6)	
C14	0.4559 (2)	0.8919 (2)	0.58461 (13)	0.0651 (7)	
H14	0.4610	0.9723	0.5692	0.078*	
C15	0.54485 (19)	0.8117 (2)	0.56344 (13)	0.0615 (6)	
H15	0.6087	0.8385	0.5331	0.074*	
C16	0.9094 (2)	0.5868 (2)	0.66041 (14)	0.0808 (8)	
H16A	0.8870	0.5157	0.6908	0.097*	
H16B	0.8450	0.6462	0.6639	0.097*	
C17	1.0262 (2)	0.6350 (3)	0.69752 (16)	0.1104 (10)	
H17A	1.0183	0.6549	0.7552	0.166*	
H17B	1.0473	0.7058	0.6676	0.166*	
H17C	1.0894	0.5757	0.6941	0.166*	
C18	0.7407 (2)	0.6940 (2)	0.37180 (15)	0.0825 (8)	
H18A	0.8159	0.6493	0.3665	0.099*	
H18B	0.7624	0.7741	0.3919	0.099*	
C19	0.6704 (2)	0.7017 (3)	0.29006 (15)	0.1278 (12)	
H19A	0.7187	0.7417	0.2509	0.192*	
H19B	0.5962	0.7461	0.2957	0.192*	
H19C	0.6504	0.6221	0.2701	0.192*	

### Atomic displacement parameters (Å^2^)

**Table:**

	*U*^11^	*U*^22^	*U*^33^	*U*^12^	*U*^13^	*U*^23^
Cl1	0.0971 (5)	0.0588 (4)	0.1362 (6)	0.0063 (4)	0.0216 (5)	−0.0038 (4)
Cl2	0.0926 (5)	0.0975 (6)	0.1076 (6)	0.0270 (4)	0.0303 (4)	−0.0125 (4)
O1	0.0454 (9)	0.0792 (11)	0.0551 (10)	−0.0086 (8)	0.0086 (7)	−0.0004 (9)
O2	0.0550 (9)	0.0737 (10)	0.0472 (9)	−0.0135 (8)	0.0046 (7)	0.0102 (8)
N1	0.0432 (10)	0.0544 (12)	0.0678 (12)	−0.0047 (10)	0.0177 (9)	0.0051 (10)
N2	0.0517 (11)	0.0586 (12)	0.0557 (11)	0.0029 (10)	0.0168 (9)	0.0101 (10)
C1	0.0485 (13)	0.0586 (15)	0.0666 (16)	−0.0042 (12)	0.0103 (12)	−0.0005 (13)
C2	0.0601 (15)	0.0582 (16)	0.0709 (16)	−0.0117 (13)	0.0111 (13)	0.0019 (13)
C3	0.0608 (15)	0.0520 (15)	0.0669 (16)	−0.0025 (13)	0.0020 (13)	−0.0105 (13)
C4	0.0563 (15)	0.0714 (18)	0.0742 (17)	0.0052 (14)	0.0118 (13)	−0.0077 (15)
C5	0.0559 (15)	0.0652 (17)	0.0620 (16)	−0.0039 (13)	0.0150 (12)	0.0014 (13)
C6	0.0417 (12)	0.0543 (15)	0.0484 (13)	−0.0077 (12)	0.0037 (11)	−0.0028 (11)
C7	0.0491 (13)	0.0641 (15)	0.0434 (13)	−0.0069 (12)	0.0065 (11)	−0.0005 (12)
C8	0.0480 (13)	0.0579 (14)	0.0474 (14)	−0.0078 (12)	0.0064 (11)	0.0014 (12)
C9	0.0435 (13)	0.0579 (15)	0.0541 (14)	−0.0041 (11)	0.0057 (11)	0.0031 (12)
C10	0.0454 (13)	0.0590 (15)	0.0399 (13)	−0.0031 (12)	0.0024 (11)	0.0001 (11)
C11	0.0515 (14)	0.0584 (15)	0.0675 (15)	−0.0062 (13)	0.0126 (12)	0.0010 (13)
C12	0.0521 (15)	0.0777 (18)	0.0721 (17)	−0.0063 (14)	0.0176 (13)	−0.0044 (15)
C13	0.0596 (15)	0.0689 (17)	0.0558 (15)	0.0058 (14)	0.0077 (12)	−0.0100 (13)
C14	0.0762 (17)	0.0588 (16)	0.0605 (15)	0.0058 (14)	0.0066 (14)	0.0058 (13)
C15	0.0601 (15)	0.0659 (17)	0.0605 (15)	−0.0007 (13)	0.0167 (12)	0.0082 (13)
C16	0.0719 (17)	0.117 (2)	0.0537 (16)	−0.0122 (16)	0.0074 (14)	−0.0114 (15)
C17	0.086 (2)	0.150 (3)	0.090 (2)	−0.005 (2)	−0.0247 (17)	−0.016 (2)
C18	0.0868 (18)	0.0938 (19)	0.0683 (18)	−0.0140 (16)	0.0153 (16)	0.0235 (15)
C19	0.131 (3)	0.195 (3)	0.0564 (18)	−0.041 (2)	−0.0022 (18)	0.041 (2)

### Geometric parameters (Å, °)

**Table:**

Cl1—C3	1.747 (2)	C9—H9A	0.9700
Cl2—C13	1.736 (2)	C9—H9B	0.9700
O1—C16	1.422 (2)	C10—C15	1.384 (3)
O1—C7	1.422 (2)	C10—C11	1.389 (2)
O2—C18	1.423 (2)	C11—C12	1.372 (3)
O2—C8	1.424 (2)	C11—H11	0.9300
N1—C6	1.380 (2)	C12—C13	1.365 (3)
N1—C7	1.437 (2)	C12—H12	0.9300
N1—C9	1.440 (2)	C13—C14	1.366 (3)
N2—C10	1.384 (2)	C14—C15	1.381 (3)
N2—C8	1.429 (2)	C14—H14	0.9300
N2—C9	1.443 (2)	C15—H15	0.9300
C1—C2	1.376 (3)	C16—C17	1.466 (3)
C1—C6	1.385 (2)	C16—H16A	0.9700
C1—H1	0.9300	C16—H16B	0.9700
C2—C3	1.366 (3)	C17—H17A	0.9600
C2—H2	0.9300	C17—H17B	0.9600
C3—C4	1.369 (3)	C17—H17C	0.9600
C4—C5	1.372 (3)	C18—C19	1.467 (3)
C4—H4	0.9300	C18—H18A	0.9700
C5—C6	1.397 (3)	C18—H18B	0.9700
C5—H5	0.9300	C19—H19A	0.9600
C7—C8	1.526 (3)	C19—H19B	0.9600
C7—H7	0.9800	C19—H19C	0.9600
C8—H8	0.9800		
			
C16—O1—C7	115.22 (15)	H9A—C9—H9B	109.1
C18—O2—C8	113.37 (16)	N2—C10—C15	122.01 (19)
C6—N1—C7	124.76 (17)	N2—C10—C11	120.6 (2)
C6—N1—C9	122.01 (16)	C15—C10—C11	117.4 (2)
C7—N1—C9	113.14 (16)	C12—C11—C10	121.1 (2)
C10—N2—C8	124.56 (17)	C12—C11—H11	119.4
C10—N2—C9	121.99 (17)	C10—C11—H11	119.4
C8—N2—C9	112.07 (16)	C13—C12—C11	120.7 (2)
C2—C1—C6	121.0 (2)	C13—C12—H12	119.7
C2—C1—H1	119.5	C11—C12—H12	119.7
C6—C1—H1	119.5	C12—C13—C14	119.4 (2)
C3—C2—C1	119.9 (2)	C12—C13—Cl2	120.12 (19)
C3—C2—H2	120.1	C14—C13—Cl2	120.5 (2)
C1—C2—H2	120.1	C13—C14—C15	120.4 (2)
C2—C3—C4	120.4 (2)	C13—C14—H14	119.8
C2—C3—Cl1	120.04 (18)	C15—C14—H14	119.8
C4—C3—Cl1	119.48 (18)	C14—C15—C10	121.0 (2)
C3—C4—C5	120.1 (2)	C14—C15—H15	119.5
C3—C4—H4	119.9	C10—C15—H15	119.5
C5—C4—H4	119.9	O1—C16—C17	108.81 (19)
C4—C5—C6	120.5 (2)	O1—C16—H16A	109.9
C4—C5—H5	119.8	C17—C16—H16A	109.9
C6—C5—H5	119.8	O1—C16—H16B	109.9
N1—C6—C1	120.87 (19)	C17—C16—H16B	109.9
N1—C6—C5	121.08 (19)	H16A—C16—H16B	108.3
C1—C6—C5	118.0 (2)	C16—C17—H17A	109.5
O1—C7—N1	115.03 (16)	C16—C17—H17B	109.5
O1—C7—C8	113.71 (17)	H17A—C17—H17B	109.5
N1—C7—C8	103.01 (15)	C16—C17—H17C	109.5
O1—C7—H7	108.3	H17A—C17—H17C	109.5
N1—C7—H7	108.3	H17B—C17—H17C	109.5
C8—C7—H7	108.3	O2—C18—C19	109.0 (2)
O2—C8—N2	109.47 (16)	O2—C18—H18A	109.9
O2—C8—C7	111.79 (16)	C19—C18—H18A	109.9
N2—C8—C7	103.88 (16)	O2—C18—H18B	109.9
O2—C8—H8	110.5	C19—C18—H18B	109.9
N2—C8—H8	110.5	H18A—C18—H18B	108.3
C7—C8—H8	110.5	C18—C19—H19A	109.5
N1—C9—N2	102.80 (16)	C18—C19—H19B	109.5
N1—C9—H9A	111.2	H19A—C19—H19B	109.5
N2—C9—H9A	111.2	C18—C19—H19C	109.5
N1—C9—H9B	111.2	H19A—C19—H19C	109.5
N2—C9—H9B	111.2	H19B—C19—H19C	109.5
			
C6—C1—C2—C3	−0.1 (3)	C9—N2—C8—C7	−21.7 (2)
C1—C2—C3—C4	0.4 (3)	O1—C7—C8—O2	138.53 (16)
C1—C2—C3—Cl1	178.08 (16)	N1—C7—C8—O2	−96.31 (17)
C2—C3—C4—C5	0.2 (3)	O1—C7—C8—N2	−103.52 (18)
Cl1—C3—C4—C5	−177.52 (17)	N1—C7—C8—N2	21.63 (19)
C3—C4—C5—C6	−1.0 (3)	C6—N1—C9—N2	179.82 (17)
C7—N1—C6—C1	178.06 (18)	C7—N1—C9—N2	3.1 (2)
C9—N1—C6—C1	1.7 (3)	C10—N2—C9—N1	179.41 (16)
C7—N1—C6—C5	−1.4 (3)	C8—N2—C9—N1	12.3 (2)
C9—N1—C6—C5	−177.81 (18)	C8—N2—C10—C15	−14.3 (3)
C2—C1—C6—N1	179.80 (19)	C9—N2—C10—C15	−179.81 (19)
C2—C1—C6—C5	−0.7 (3)	C8—N2—C10—C11	167.52 (18)
C4—C5—C6—N1	−179.23 (19)	C9—N2—C10—C11	2.0 (3)
C4—C5—C6—C1	1.3 (3)	N2—C10—C11—C12	177.02 (19)
C16—O1—C7—N1	−50.9 (2)	C15—C10—C11—C12	−1.2 (3)
C16—O1—C7—C8	67.6 (2)	C10—C11—C12—C13	0.3 (3)
C6—N1—C7—O1	−68.0 (2)	C11—C12—C13—C14	0.4 (3)
C9—N1—C7—O1	108.68 (19)	C11—C12—C13—Cl2	179.42 (17)
C6—N1—C7—C8	167.75 (18)	C12—C13—C14—C15	0.0 (3)
C9—N1—C7—C8	−15.6 (2)	Cl2—C13—C14—C15	−179.05 (16)
C18—O2—C8—N2	160.47 (17)	C13—C14—C15—C10	−1.0 (3)
C18—O2—C8—C7	−85.0 (2)	N2—C10—C15—C14	−176.62 (19)
C10—N2—C8—O2	−68.9 (2)	C11—C10—C15—C14	1.6 (3)
C9—N2—C8—O2	97.87 (19)	C7—O1—C16—C17	−170.4 (2)
C10—N2—C8—C7	171.58 (17)	C8—O2—C18—C19	−174.5 (2)
